# Prognostic Factors Affecting Long-Term Survival after Resection for Noncolorectal, Nonneuroendocrine, and Nonsarcoma Liver Metastases

**DOI:** 10.1155/2017/5184146

**Published:** 2017-07-24

**Authors:** Fabio Uggeri, Enrico Pinotti, Marta Sandini, Luca Nespoli, Luca Gianotti, Fabrizio Romano

**Affiliations:** Department of Medicine and Surgery, University of Milano-Bicocca, Via Cadore 48, 20900 Monza, Italy

## Abstract

**Aim:**

To evaluate feasibility and long-term outcome after hepatic resection for noncolorectal, nonneuroendocrine, and nonsarcoma (NCNNNS) liver metastases in a single center.

**Methods:**

We retrospectively reviewed our experience on patients who underwent surgery for NCNNNS liver metastases from 1995 to 2015. Patient baseline characteristics, tumor features, treatment options, and postoperative outcome were retrieved.

**Results:**

We included 47 patients. The overall 5-year survival (OS) rate after hepatectomy was 27.6%, with a median survival of 21 months. Overall survival was significantly longer for patients operated for nongastrointestinal liver metastases when compared with gastrointestinal (41 versus 10 months; *p* = 0.027). OS was significantly worse in patients with synchronous metastases than in those with metachronous disease (10 versus 22 months; *p* = 0.021). The occurrence of major postoperative complication negatively affected long-term prognosis (OS 23.5 versus 9.0 months; *p* = 0.028). Preoperative tumor characteristics (number and size of the lesions), intraoperative features (extension of resection, need for transfusions, and Pringle's maneuver), and R0 at pathology were not associated with differences in overall survival.

**Conclusion:**

Liver resection represents a possible curative option for patients with NCNNNS metastases. The origin of the primary tumor and the timing of metastases presentation may help clinicians to better select which patients could take advantages from surgical intervention.

## 1. Introduction

Liver metastasis is the most common indication for hepatic surgery. Among those, colorectal metastases represent the most frequent, showing a 5-year overall survival (OS) ranging from 25% to 47% [1–4]. Also, for neuroendocrine and sarcoma metastatic neoplasms, surgical treatment is considered a suitable chance for cure, with 5-year survival between 36% and 86% [5–8].

Conversely, the role of surgery for noncolorectal, nonneuroendocrine, and nonsarcoma (NCNNNS) liver metastatic disease is less defined, due to scarcity of data and contrasting results [9–11]. Adam et al. [12] in a large retrospective multicenter study reported that patients undergoing liver resection for NCNNNS metastases experienced an overall survival of 36% and 23%, at 5 and 10 years, respectively. These data have been recently confirmed in a review reporting 5- and 10-year survival rates up to 42% and 25%, respectively [13].

Adam and colleagues [12] suggested a clinical and pathological score to estimate OS after resection for NCNNNS; nonetheless, the large heterogeneity of patients included in these series and validation cohorts prevents to clearly define who really benefit from a surgical approach.

With our retrospective single-center series, we aimed to find potential predictive factors able to modulate OS in patients operated for noncolorectal, nonneuroendocrine, and nonsarcoma liver metastases.

## 2. Materials and Methods

From a prospective maintained database, we retrospectively retrieved data on adult patients who underwent liver resection for NCNNNS from 1995 to 2015. Exclusion criteria were extrahepatic disease at preoperative radiologic workup and American Society of Anesthesiologists (ASA) class > 3.

Local ethical committee's review of the protocol deemed that formal approval was not required owing to the retrospective, observational, and anonymous nature of this study.

Stage and disease extension were assessed at total body contrast-enhanced CT scan or at magnetic resonance imaging. Contrast-enhanced ultrasound and positron emission tomography were used in case of uncertainty to further exclude extrahepatic dissemination. All patients underwent intraoperative ultrasound to confirm technical resectability.

### 2.1. Patient Baseline, Tumor Characteristics, and Intraoperative and Pathological Features

Age, gender, and comorbidity were accrued. Intraoperative parameters considered were the rate of Pringle's maneuver, the need for blood transfusion, and the extension of hepatic resection. According to the International Hepato-Pancreato-Biliary Association [14], major hepatectomy was defined as liver resection equal or more than three segments.

We collected timing of metastasis appearance, number and size of lesions, and radical resection (R0) at pathology. The site of the primary neoplasm was subgrouped into gastrointestinal (esophagus, stomach, pancreas, and gallbladder) and nongastrointestinal origin (breast, lung, kidney, ovary/uterus, and skin melanoma).

Variables potentially affecting OS were selected on the basis of clinical plausibility and on previous results [11, 12, 15–19].

### 2.2. Primary and Secondary Endpoints

Occurrence and severity of all postoperative complications were defined according to the Clavien-Dindo (CD) classification [20]. In each patient, the highest CD grade complication was considered.

The primary endpoint was the 5-year overall patient survival. Follow-up was carried out by office visits, blood level of tumor markers, and radiologic imaging. Abdominal ultrasound scan was prescribed every three months for the first two years, while contrast-enhanced computed tomography (CT) scan was added in case of abnormal findings. The frequency of radiologic, laboratory, and clinical follow-up was every six months after the second year.

As secondary endpoint, we aimed to externally validate the Adam score (12). As proposed by Adam and coworkers, we assigned points as the following: 1 point for major resection; 1 point for age between 30 and 60 years; 2 points for age more than 60 years; 1 point for a disease-free survival period ranging from 12 to 24 months; 2 points for a disease-free survival interval shorter than 12 months; 0 points for metastatic breast cancer; 2 points for squamous tumors; 3 points for melanoma; and 1 point for all other sites and histology.

### 2.3. Statistical Analysis

All statistical computations were performed using IBM SPSS, version 22 (IBM Corp., Armonk, NY). Continuous variables were expressed as median and range. Categorical variables were presented as absolute numbers and percentages. Survival analysis was performed with the Kaplan-Meier method. The difference in survival for subgroup of patients, according to the categorical values and the dichotomized continuous values, was tested with the log-rank (Mantel-Cox) test. A two-sided *p* value <0.05 was considered significant.

## 3. Results

**Figure 1:**
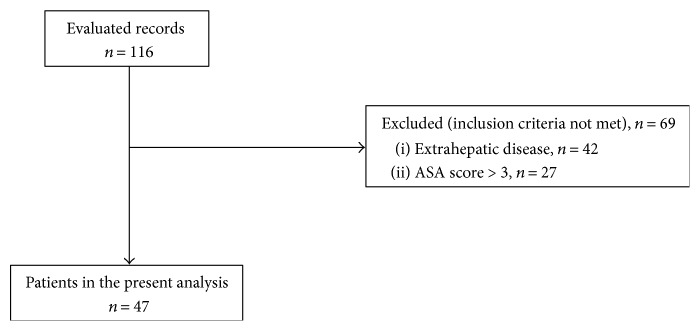
Patients included in the study.

We evaluated overall 116 records for inclusion and exclusion criteria. Sixty-nine (59.5%) patients were further excluded: 42 patients for the presence of extrahepatic disease and 27 patients because of ASA score > 3 (Figure 1). Forty-seven patients were finally included for the analysis. The mean follow-up was 60 months (range 4–211).

Table 1 depicts baseline patient features, tumor characteristics, and surgical details. The median size of metastasis was 2.4 cm (range 0.5–12 cm). Patients presented with single lesion in 29 cases out of 47 (61.7%) and 5 patients (10.6%) had tumor diameter larger than 5 cm. The median time of Pringle's maneuver was 30 min (range 15–55 min).

Primary tumors were from GI origin in 25 patients (53.2%), mostly from gastric cancers. Among nongastrointestinal (non-GI) origin sites of primary tumor were the kidney (12.8%), breast (10.6%), ovary/uterus (10.6%), and melanoma (8.5%), as shown in Table 2.

Major complications, defined as CD ≥ 3, occurred in 5 patients (10.6%); 4 patients experienced perihepatic collections and needed radiological percutaneous drainage, and 1 patient underwent relaparotomy for ventral hernia; minor complications occurred in 6 patients. Fifteen patients (31.9%) needed blood transfusion during admission. We observed no postoperative mortality. During follow-up period, no patients were submitted to repeated hepatic resection in case of disease relapse.

Mean length of in-hospital stay was 11 days (range 6–25).

### 3.1. Survival Analysis

**Figure 2:**
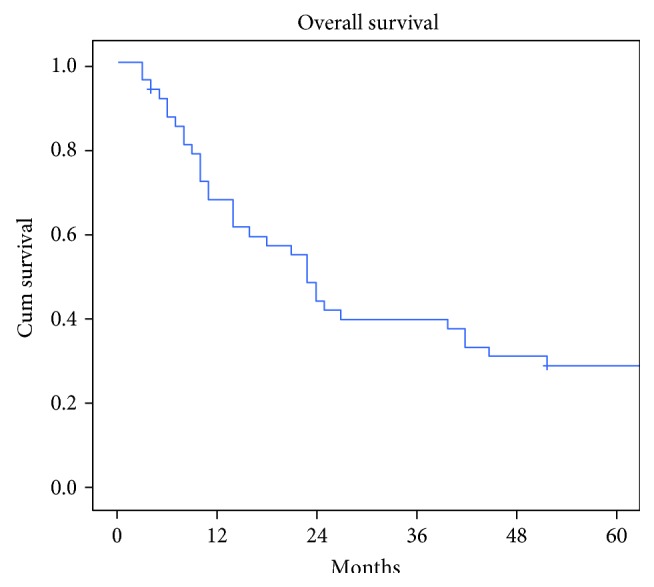
Overall survival.

**Figure 3:**
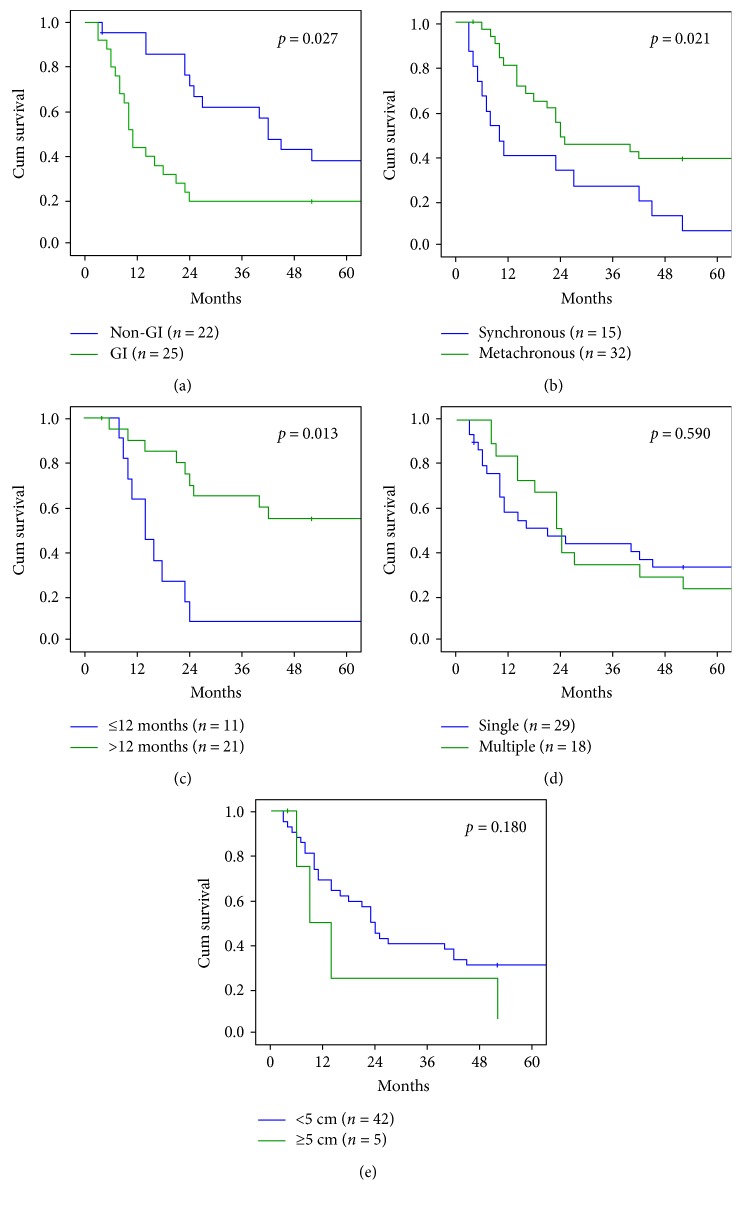
Overall survival according to tumor characteristics. (a) Gastrointestinal versus nongastrointestinal. (b) Synchronous versus metachronous. (c) Metachronous ≤ 12 months versus metachronous > 12 months. (d) Single versus multiple lesions. (e) Single lesions ≥ 5 cm versus <5 cm.

The overall 5-year survival rate was 27.6%, with a median survival of 21 months (range 4–211) (Figure 2()). The survival rate was 61.7% at 1 year, 42.5% at 2 years, 38.2% at 3 years, and 29.7% at 4 years. Median overall survival was significantly better in patients operated for non-GI liver metastases (41 months, range 4–211) compared with GI origin (10 months, range 4–137) (*p* = 0.027 at log-rank test). The rates of 1-, 3-, and 5-year survival were 40.0%, 20%, and 16% for GI liver metastases and 86.3%, 59.0%, and 37.5% for non-GI liver metastases (Figure 3()(a)), respectively. Liver resection for pancreatic cancer metastases showed the worse prognosis (median OS 6 months; range 4–8 months), followed by gallbladder and esophagus cancer (median OS 8 months, range 6–10) and gastric cancer (median OS 15 months, range 6–137 months). For non-GI origin, we observed a median OS of 68 months (range 11–211) for renal cell cancer, 90 months for breast cancer (range 20–106), 37 months for gynecological cancer (range 4–75), 31.5 months for melanoma (range 22–84), and 19 8 months for lung cancer (range 14–25).

Fifteen patients (31.9%) out of 47 underwent liver resection for synchronous and 32 (68.1%) for metachronous liver metastases. Patients with metachronous metastases showed significantly better prognosis, with an OS of 22 months (range 4–211), when compared with synchronous (OS 10 months, range 4–113; *p* = 0.021 at log-rank test) (Figure 3()(b)).

At a further analysis of metachronous disease, the median survival for liver metastases occurred before than 1 year from surgical resection of primary tumor was 14 months (range 8–137) versus 51 months (range 4–211), in case of relapse after 12 months (*p* = 0.013 at log-rank test) (Figure 3(c)).

Patients operated for a single liver metastasis showed a trend in improved OS, when compared with those with multiple metastases: median OS was 14 versus 22 months, respectively (*p* = 0.590 at log-rank test) (Figure 3(d)).

Similarly, the size of the metastasis did not affect survival time. The median survival in patients with metastases ≥5 cm was 14 months (range 4–52) versus 22 months (range 4–211) in patients with lesions <5 cm (*p* = 0.180 at log-rank test) (Figure 3(e)).

### 3.2. Survival according to Operative Characteristics

**Figure 4:**
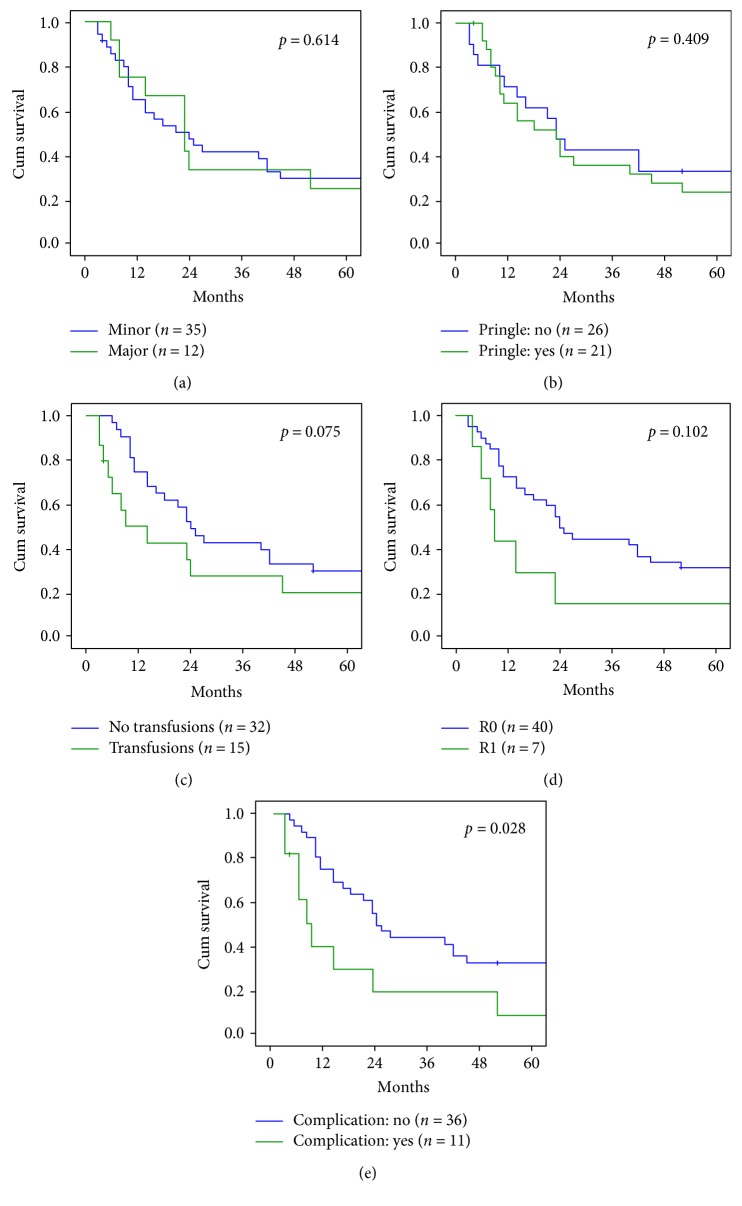
Overall survival according to surgical and oncologic characteristics. (a) Minor versus major resection. (b) Pringle's maneuver: yes versus no. (c) Transfusion: yes versus no. (d) Resection margins R0 versus R1. (e) Postoperative complication: yes versus no.

In 12 patients (25.5%), we performed a major hepatectomy and in 35 patients (74.5%) a minor resection. There was no significant difference in terms of long-term survival, with a median overall survival of 21 months for both major and minor resections (*p* = 0.614 at log-rank test) (Figure 4(a)).

Median survival time was 23 months (range 4–211) in patients who received Pringle's maneuver and 16 (range 4–137) in those who did not received (*p* = 0.409 at log-rank test) (Figure 4(b)). Patients who need blood transfusion had a worse prognosis with a median overall survival of 9 months (range 4–106), when compared with not transfused (23.5 months, range 6–211; *p* = 0.075 at log-rank test) (Figure 4(c)).

Positive resection margin (R1) was reported in 7 cases (14.9%). Although the median survival was 9 months (range 4–86) in R1 patients versus 22.5 months (range 4–211) in R0 resections, we did not observe a statistically relevant difference (*p* = 0.102 at log-rank test) (Figure 4(d)).

The occurrence of major postoperative complications (Clavien-Dindo ≥ 3) significantly affected overall survival. Actually, uncomplicated patients had a median OS of 23.5 9 months (range 4–211), while complicated patients had median OS of 9 months (range 4–118; *p* = 0.028 at log-rank test) (Figure 4(e)).  Table 3 describes our 5-year survival rate, according to the computed Adam score and the corresponding expected survival rate. With the limitation of the small sample size, we observed a similar trend between observed and estimated survival rates.

## 4. Discussion

The present data on long-term results of patients undergoing liver resection for NCNNNS liver metastases are consistent with previous findings [15, 16, 21, 22] reporting an overall 5-year survival rate ranging from 19% to 40%. Given this wide range, there might be space for further improvement by optimizing patient selection and accordingly offer surgery to subjects that may gain the greatest benefit from hepatic resection with curative intent. In this line of thought, we reviewed our experience to identify potential risk factors for poor prognosis and thus addressing patients to alternative treatments.

The first significant observation was that patients with liver metastases from non-GI primary tumors have a better prognosis than those with metastatic disease from GI origin tumors as shown by others [9, 16, 18, 20, 22] with the exception of metastases from gastric cancer, which have an acceptable prognosis after radical liver resection [23–25]. Among the non-GI cancers, the best prognosis was observed in breast and kidney cancers followed by ovarian and melanoma metastatic cancers, while lung tumors showed the worst overall survival.

Based on our observations, another key factor affecting long-term outcome is the time of metastasis appearance as previously suggested [15, 20, 26]. In fact, survival of patients with synchronous metastases was significantly shorter than those with metachronous metastases. Yet, among the subjects with metachronous presentation of hepatic disease, there was a remarkable difference in survival when the metastasis manifestation was within 12 months from the operation for the primary tumor. This event changed radically the prognosis with an overlapping outcome of synchronous disease. The current and other results suggest that timing of metastasis presentation should be considered as an important surrogate of the biologic aggressiveness of the disease.

We observed a similar prognosis in patients who underwent liver resection for single or multiple metastases and in patients receiving a major or minor hepatic resection. Previous results on the role of extension of surgical resection and number of lesions as prognostic factors are contradictory with reports showing a significant negative impact of survival [11, 12, 19, 22] and others with results disclosing no effect [15, 21, 27, 28].

Our experience confirms earlier findings [29, 30] showing that clamping of hepatic pedicle was not associated with any significant variation of the long-term survival. Similarly, the need of blood transfusion did not affect prognosis confirming the results of other authors [27, 31]. Achieving a radical resection (R0) is one of the most important target to influence the prognosis after hepatic resection [12, 16, 32, 33]. The present results validate this cornerstone of surgical oncology, even though for a possible type II error, we did not reach a significant difference in long-term survival between patients with microscopic margin infiltration (median 9 months) and those with radical operation (median 22.5 month). The role of postoperative morbidity in long-term prognosis and recurrence in cancer patients is debated [11, 34, 35]. One study [36] in over 105000 patients found a strong association between long-term survival following surgery and the occurrence of postoperative complications. In fact, among patients with postoperative morbidity compared with that in those without, the 5-year survival rate was reduced to one-third. This correlation was further confirmed by a recent meta-analysis [37, 38]. However, other authors [8, 16] have failed to find an association between complications and long-term survival. Our results are consistent with the hypothesis that postoperative morbidity is one of the determinant factors for poor long-term results, although the mechanism by which complications affect survival remains uncertain.

Adam et al. [12] proposed a preoperative scoring system, based on patient-related and tumor-related characteristics, to estimate long-term outcome of patients with noncolorectal and nonneuroendocrine metastatic disease. They included secondary liver lesions from sarcoma that we did not take into account. With this limitation and the small number of patients of our series, the present results suggest that the Adam metric is reliable in predicting survival and should be used to select patients whom to offer surgery with potentially curative intent.

## 5. Conclusion

The findings presented in this study suggest that before proposing a liver resection for NCNNNS metastases, it is essential to consider preoperatively the origin of the primary tumor and the timing of metastasis appearance. Major hepatectomy, even associated with Pringle's maneuver and use of blood transfusion, may be safely done without affecting long-term outcome and should be carry out with the target of accomplishing an R0 resection. Given the relevant effect of postoperative morbidity on outcome, such major operations should be performed in referral centers or by experienced surgeons.

## Figures and Tables

**Table 1:** Baseline patient features, tumor characteristics, and surgical details.

Age	69	37–82
Gender		
Male	21	44.7%
Female	26	55.3%
Presentation		
Synchronous	15	31.9%
Metachronous	32	68.1%
Metachronous ≤ 12 months	11	23.4%
Metachronous > 12 months	21	44.6%
Number of lesions		
Single	29	61.7%
Multiple	18	38.3%
Size of lesion(s)		
≥5 cm	5	10.6%
<5 cm	42	89.4%
Type of resection		
Major	12	25.5%
Left hepatectomy	6	12.7%
Trisegmentectomy	3	6.4%
Right hepatectomy	2	4.2%

**Table 2:** Primary tumor histology.

Gastrointestinal primary site (*n* = 25)		
Stomach	17	36.2%
Pancreas	3	6.4%
Gallbladder	3	6.4%
Esophagus	2	4.3%
Nongastrointestinal primary site (*n* = 22)		
Kidney	6	12.8%
Breast	5	10.6%
Ovary/uterus	5	10.6%
Melanoma	4	8.5%
Lung	2	4.3%

**Table 3:** Comparison with Adam score.

Adam score	Number of patients	Actual 5-year survival	Expected 5-year survival according to Adam score
2	1	100%	45%
3	2	50%	36%
4	13	53%	27%
5	22	13%	19%
6	7	0%	12%
7	0	—	7%
8	2	0%	4%

## Additional Points

*Institutional Review Board Statement*. Under the Italian law, anonymous retrospective studies do not require approval by the ethics committee and they do not require a patient's consent.

## Data Access

Statistical code and dataset are available from the corresponding author [fabio.uggeri@unimib.it]; no other data are available.
